# Establishing a machine learning model for predicting nutritional risk through facial feature recognition

**DOI:** 10.3389/fnut.2023.1219193

**Published:** 2023-09-15

**Authors:** Jingmin Wang, Chengyuan He, Zhiwen Long

**Affiliations:** ^1^College of International Engineering, Xi’an University of Technology, Xi’an, China; ^2^Recovery Plus Clinic, Chengdu, China

**Keywords:** support vector machine, U-net, histogram of oriented gradient, nutrition, NRS-2002

## Abstract

**Background:**

Malnutrition affects many worldwide, necessitating accurate and timely nutritional risk assessment. This study aims to develop and validate a machine learning model using facial feature recognition for predicting nutritional risk. This innovative approach seeks to offer a non-invasive, efficient method for early identification and intervention, ultimately improving health outcomes.

**Methods:**

We gathered medical examination data and facial images from 949 patients across multiple hospitals to predict nutritional status. In this multicenter investigation, facial images underwent preprocessing via face alignment and cropping. Orbital fat pads were isolated using the U-net model, with the histogram of oriented gradient (HOG) method employed for feature extraction. Standardized HOG features were subjected to principal component analysis (PCA) for dimensionality reduction. A support vector machine (SVM) classification model was utilized for NRS-2002 detection. Our approach established a non-linear mapping between facial features and NRS-2002 nutritional risk scores, providing an innovative method for evaluating patient nutritional status.

**Results:**

In context of orbital fat pad area segmentation with U-net model, the averaged dice coefficient is 88.3%. Our experimental results show that the proposed method to predict NRS-2002 scores achieves an accuracy of 73.1%. We also grouped the samples by gender, age, and the location of the hospital where the data were collected to evaluate the classification accuracy in different subsets. The classification accuracy rate for the elderly group was 85%, while the non-elderly group exhibited a classification accuracy rate of 71.1%; Furthermore, the classification accuracy rate for males and females were 69.2 and 78.6%, respectively. Hospitals located in remote areas, such as Tibet and Yunnan, yielded a classification accuracy rate of 76.5% for collected patient samples, whereas hospitals in non-remote areas achieved a classification accuracy rate of 71.1%.

**Conclusion:**

The attained accuracy rate of 73.1% holds significant implications for the feasibility of the method. While not impeccable, this level of accuracy highlights the potential for further improvements. The development of this algorithm has the potential to revolutionize nutritional risk assessment by providing healthcare professionals and individuals with a non-invasive, cost-effective, and easily accessible tool.

## 1. Introduction

Disease-related malnutrition is a multifactorial condition that can result from a combination of factors, including reduced food intake due to poor appetite, chewing or swallowing difficulties, gastrointestinal symptoms such as nausea and vomiting, and disease-related factors such as inflammation, altered metabolism, and increased nutrient losses (1). In addition to inadequate food intake, increased nutrient needs owing to metabolic stress or catabolic states, such as cancer or severe illness, can also contribute to disease-related malnutrition. Malnutrition can result in a variety of adverse health effects, including poor immunological function, muscular loss, slowed wound healing, and an increased risk of infection and mortality (2).

Consequently, screening for malnutrition is a vital component of patient care, since it identifies patients at risk of malnutrition and enables the provision of appropriate therapies to prevent or cure it (3). Early warning of malnutrition enables early nutritional therapy, such as oral nutritional supplements, enteral or parenteral feeding, or dietary adjustments, which can improve health outcomes, minimize the risk of complications, and lower healthcare costs (4). In addition, screening for malnutrition allows healthcare providers to observe changes in a patient’s nutritional status over time, allowing for continuing monitoring and updates to the nutritional plan (2). Using a screening tool can therefore be an efficient method for identifying and treating malnutrition in hospital and outpatient settings.

Nutritional risk screening (NRS-2002) is a validated tool that can be used in both inpatient and outpatient to screen for malnutrition risk (5). This tool evaluates many risk variables for malnutrition, including age, weight loss, body mass index, food consumption, and illness severity. It has been shown that the NRS-2002 is a reliable and accurate instrument for detecting patients at risk of malnutrition in a variety of subjects and demographics (6). Many professional organizations, including the European Society for Clinical Nutrition and Metabolism (ESPEN) and the American Society for Parenteral and Enteral Nutrition (ASPEN), have recommended its usage (7).

The insufficiency of assessment rates for the nutritional risk screening (NRS-2002) in China poses a significant challenge in identifying malnourished patients (8). Moreover, when patients return to their homes after being discharged from the hospital, self-assessment of nutritional status becomes difficult (9). It is proposed that exploring facial recognition technology as a potential solution for determining whether a patient is experiencing malnutrition could be beneficial.

Facial features have been integral to the evaluation of malnutrition, as demonstrated by their inclusion in tools such as the Patient-Generated Subjective Global Assessment (PG-SGA) (10). In the context of the PG-SGA, facial features serve as crucial indicators of muscle and fat loss. Nevertheless, the reliance on subjective evaluations introduces inconsistencies in assessment criteria, leading to issues in reliability and validity. Incorporating artificial intelligence (AI) in analyzing facial features and their association with nutritional risk could help mitigate these discrepancies.

By employing AI algorithms to establish a consistent standard, this approach can offer numerous advantages. These include improved objectivity and reliability in malnutrition assessment, enhanced accessibility for patients, real-time monitoring and analysis of patient data, and increased efficiency for healthcare providers. Furthermore, AI-based facial recognition technology can provide a non-invasive assessment method that reduces patient discomfort and facilitates regular evaluation, which is essential for monitoring and adjusting nutritional interventions.

Although previous research has attempted to develop AI models capable of predicting nutritional status based on facial images (11), these studies have not yet connected these AI models with clinically recognized nutritional assessment tools such as the NRS-2002. This gap in the literature emphasizes the need for further investigation in this area.

To address the limitations of existing research and enhance malnutrition assessment, we plan to focus on collecting an extensive dataset of facial images and NRS-2002 scores in China. Through the application of machine learning techniques, we aim to establish a non-linear mapping relationship between facial features and NRS-2002 scores, substantiating the feasibility of this approach.

Our study will contribute to the field in several ways. First, we will provide evidence for the effectiveness of AI-based facial recognition in identifying patients at risk of malnutrition. Second, we will develop a more objective, reliable, and accessible malnutrition assessment method that can be implemented in various settings, including hospitals and homes. Third, we will offer valuable insights into the unique challenges and needs of the Chinese population concerning malnutrition assessment and treatment. Lastly, our research will pave the way for the development of additional AI-based assessment tools that can address other health-related issues beyond malnutrition.

In addition to these contributions, our study will have significant practical implications. The successful implementation of AI-based facial recognition technology in malnutrition assessment could lead to improved health outcomes for patients at risk of malnutrition, as well as a reduction in associated healthcare costs. Moreover, our research could inspire the development of user-friendly applications that facilitate patient self-assessment, empowering individuals to take control of their nutritional health.

In conclusion, we hypothesize that integrating AI-based facial recognition technology into malnutrition assessment holds the potential to overcome the limitations of current methods, ultimately leading to better health outcomes for patients at risk of malnutrition. By addressing these challenges and exploring innovative solutions, our research aims to contribute to the broader goal of improving healthcare quality and accessibility for patients worldwide.

## 2. Materials and methods

### 2.1. Sample description

We utilized data collected via the R+ Clinic’s internet platform to predict patients’ nutritional status. The data, comprising medical examination information and facial images of 949 patients from various hospitals, underwent rigorous ethical review. Our aim was to develop an innovative approach for assessing patients’ nutritional status by leveraging this data. The study was sanctioned by the institutional review boards of all participating hospitals, as evidenced by the clinical trial registration numbers ChiCTR2200063512 and ChiCTR2100051648.

To ensure the precision of our analysis, we implemented stringent data filtering. We excluded samples where the patients’ face images were obscured by face masks, excessively blurry, or contained mismatched information. Additionally, we discarded data where the facial images featured glasses or were of poor quality, to accurately segment the orbital fat pad area. Through this meticulous filtering process, we eliminated 434 samples, resulting in a robust dataset of 515 high-quality samples for our study.

Filtered dataset include the clinical characteristics of 515 patients which were evaluated from 2 April 2021 to 6 June 62022 from different departments of different hospitals. We conducted data preprocessing using Python version 3.8. Subsequently, we utilized the Pandas library to compute summary statistics for the relevant variables, including means, medians, and quartiles. The source hospitals and departments of the sample are shown in Table 1. The sample population include 317 men (61.5%) and 198 women (38.4%). Based on the World Health Organization’s definition of older adults, individuals aged 65 and order are classified as the elderly group, while those under 65 are classified as the non-elderly group. There were 430 individuals (83.5%) in the non-elderly group and 85 individuals (16.5%) in the elderly group. The median age is 50 years (range, 19 to 84 years), and 25% of patients were 65 years or older.

**TABLE 1:** Statistics of samples collected from different hospitals and departments.

Hospital	Department	Frequency
Total		*N* = 515
Yunnan Cancer Hospital	NST	59
West China Hospital	Oncology	73
	Lung cancer center	52
	Biotherapy	27
	Cardiovascular medicine	11
	Thoracic surgery	1
	Else	157
Sichuan University Huaxi Hospital Tibet Chengban Branch	General surgery	15
	Endocrinology	83
	ICU	7
	Gynecology	4
	Cadre medicine	8
	Orthopedics	8
	Neurosurgery	5
	Digestive	1
	Cardiovascular medicine	3
	Ophthalmology	1

In the sample population, the NRS-2002 score ranged from a minimum of 0 to a maximum of 5. The patients are considered malnourished when NRS-2002 score is ≥3 (5). Therefore, our sample data are grouped as NRS-2002 scores greater than or equal to 3 and NRS-2002 scores less than 3. To investigate the relationship between two categorical variables, we used Python 3.8 to conduct the chi-square test with a significance level of 0.05. A *P*-value < 0.05 indicated a statistically significant association between the variables. More detailed patient baseline characteristics, stratified by NRSs, are shown in Table 2. Due to the presence of comorbidities in the sample, the total number of individuals categorized by disease exceeds 515. We categorized the age variable into two groups: 19–64 years and 65–84 years, also we classified NRS scores into two groups: scores less than 3 and scores equal to or greater than 3. We then employed the chi-square tests to determine the significance of age grouping and NRS score grouping. Similarly, we employed the chi-square tests to determine the significance of gender grouping and NRS score grouping.

**TABLE 2:** Baseline characteristics.

Parameters	Stratified according to NRS		*P*-value
	NRS <3	NRS ≥3	
N	392	123	
**Age groups**			
19–64 years	332	97	0.169
65–84 years	60	26	
**Gender groups**			
Male	240	77	0.867
Female	152	46	
**Disease**			
Cardiovascular disease	79	14	
Metabolic disease	89	13	
Cancer	136	75	
Not diagnosed	130	21	
Else	14	7	

### 2.2. Proposed methodology

**FIGURE 1:**
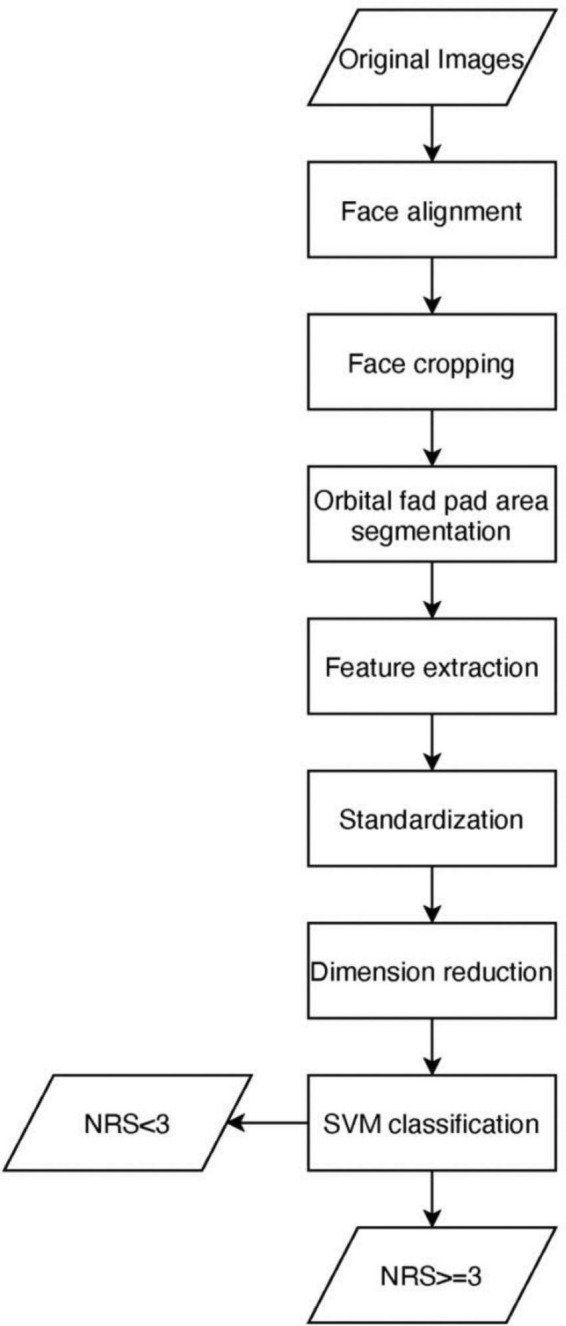
A general block diagram of NRS-2002 score detection.

**FIGURE 2:**
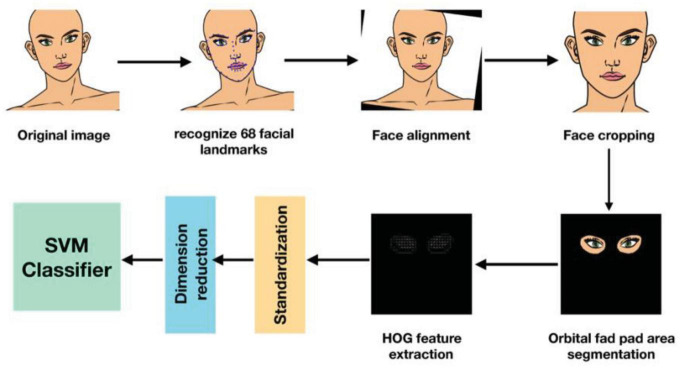
Illustration example of detection of NRS score.

The proposed methodology for NRS-2002 score detection is discussed in this section. A brief conceptual block diagram of the method is illustrated in Figure 1. As an initial stage, the face images are first preprocessed and then the orbital fat pads are segmented out using the U-net model. Next, texture features based on the Histogram of Oriented Gradients (HOG) are extracted from the orbital fat pad region in low dimensions. Thereafter, the extracted features are utilized to construct a unidimensional vector representation, which is then supplied as input to an SVM classifier, with the aim of performing NRS-2002 score classification. To describe it more explicitly, we use a cartoon face image to simulate the process of detecting NRS scores, as shown in Figure 2.

#### 2.2.1. Orbital fat pad area segmentation

##### 2.2.1.1. Face alignment

After integrating face images of the sample, we need to align the face. We utilize dlib to extract features from faces. dilb is a modern C++ toolkit that includes machine learning algorithms and tools that provide face detection algorithms and feature point algorithms (12). We use dlib face detector to recognize 68 facial landmarks of the human face.

After detecting 68 facial landmark points, here are the steps for aligning the face in an image to make sure the line connecting the eyes is horizontal: (1) Compute the average of all the landmark points located within the left and right eye regions separately, to determine the coordinates of the left and right eye center points; (2) Calculate the angle θ between the line connecting the left and right eye center coordinates and the horizontal direction; (3) Calculate coordinates of mid-point between the left and right eyes; (4) Rotate the image counterclockwise by θ with the mid-point between the left and right eyes as the base point; (5) Correspondingly, rotate each facial landmark points counterclockwise by θ, with the center coordinates between the left and right eyes as the base point.

##### 2.2.1.2. Face cropping

To reduce noise and background information, cropping the facial area of an image is necessary. The procedures are as follows.

(1) determine the central point on the face by calculating the midpoint between the leftmost and rightmost landmark points; (2) compute the center points of the eyes and mouth by taking the average of all the landmark points within their respective regions.; (3) center the face on the *X*-axis according to the central point; (4) adjust the vertical position of the face by placing the eye center point at 30% from the top of the image and the mouth center point at 35% from the bottom of the image; (5) resize the image size to 300 × 300 (13).

##### 2.2.1.3. Segmentation model

**FIGURE 3:**
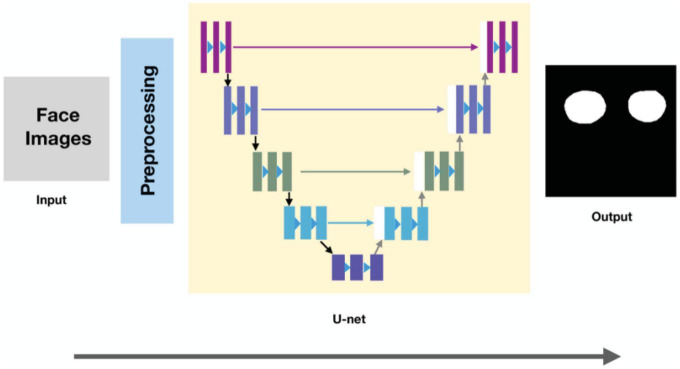
Process of extracting the region of orbital fat from face images.

The fully convolutional networks (FCN) have made substantial progress in the field of semantic segmentation of images (14). The FCN model introduces an encoder–decoder-style restoration structure as the core of the model (15). The encoder is employed to extract features, while the decoder restores as much image resolution as possible while combining high-level semantic and low-level spatial information (16). However, FCN still have some drawbacks about the asymmetry of the skip-connection and up-sampling. Based on FCNs, Ronneberger et al. (17) proposed the convolutional network called U-net for biomedical image segmentation in 2015, which is modified and extended the architecture of FCN to work with very few training images and yields more precise segmentations. Therefore, we utilized the U-Net model as a basis for the Orbital fat pad area segmentation model. The process of orbital fat pad segmentation is shown in Figure 3.

#### 2.2.2. Feature extraction

##### 2.2.2.1. Histograms of oriented gradients (HOG)

We utilize the approach of Dalal and Triggs (18) proposed that using well-normalized dense histograms of oriented gradients (HOG) to model the appearance of the orbital fat pad region. HOG is a feature base descriptor that used in image processing and computer vision to detect the objects (19). The distribution of local intensity gradients or edge direction can well characterize the local appearance and shape of objects.

The first step of HOG extraction is to compute the gradient. Then derive the orientation histogram from the orientations and magnitudes. Divide the image into cells and group cells into larger and spatially connected blocks. Construct a histogram of orientation gradient orientations binned into B bins for each cell (*B* = 9) and group several cells together to achieve normalization. A feature vector is obtained within each overlapping block of cells by sampling the histograms of the contributing spatial cells; The feature vectors of all overlapping blocks are concatenated to generate the final feature vector, and are fed into the classifier (20).

Each extracted orbital fat pad image is converted to grayscale image and normalized before feature extraction. Our choice of HOG parameters is as follows. Each block consists of 4 cells and each cell has 8 ×8 pixels. In each cell the orientation histogram has 9 bins, which correspond to orientations $i\times \frac{\pi }{9}$, *i* = 0,1,2⋯8. Consequently, each block contains 4×9=36 features and dimension of hog features per 300×300 image is 46,656.

##### 2.2.2.2. Standardization

Several machine learning algorithms tend to exhibit improved performance or faster convergence when the features are approximately normally distributed or have a similar scale. StandardScaler() transforms the dataset so that the mean of the resulting distribution is zero and the standard deviation is 1 (21). It is implemented in scikit-learn under pre-processing. Standard scores (also called *z* scores) of the samples are calculated using the formula: $z=\frac{x-\mu }{\sigma }$, where μ is the mean and σ is the standard deviation (22).

##### 2.2.2.3. Dimensionality reduction

We use principal component analysis to perform hog feature dimensionality reduction, Principal component analysis (PCA) is a method for reducing the dimensionality of such datasets while optimizing interpretability and minimizing data loss. It does so by generating new variables that are uncorrelated and successively maximize variance (23). The primary purpose of PCA is to obtain a non-redundant set of variables for a concise description of particular processes or phenomena (dimensionality reduction), which means PCA is used to extract “relevant” information from high-dimensional data sets by focusing only on those principal components that explain sufficiently high proportions of the data set’s spread or variance (24). The benefit of PCA include it can reduce the need for capacity and memory, and increases the efficiency of processes taking place in smaller dimensions (25). We apply PCA to reduce the feature dimension to 100.

#### 2.2.3. NRS-2002 score classification

In this section, we provide a comprehensive account of the feature classification module’s specifics. The purpose of this classification module is to classify the NRS-2002 score of given images of orbital fat pad into one of the two classes (NRS-2002 score greater than 3 and NRS-2002 score less than 3), depending on the features extracted from the orbital fat pad area.

Instead of using the principle of empirical risk minimization, the support vector machine (SVM) uses the principle of structural risk minimization, which aim to minimize the upper bound on the generalization error, thereby ensuring a rigorous approach to model selection and resist overfitting of data (26).

Standard SVMs were originally created for dichotomous classification tasks (i.e., binary classification problems with two classes) (26). Thereby, the main goal of SVM learning is to identify the best hyperplane that is orientated as far as possible from the closest data points from each of the classes. The training data are given *x*_1_,*x*_2_,*x*_3_,⋯,*x*_*n*_ where *X*⊆*R^d^* is a feature vector, and their labels *y*_1_,*y*_2_,*y*_3_,⋯,*y*_*n*_ that *y*_*i*_ ∈ −1,1, where *x_i_* is the vector, *y_i_* is the class label. Therefore, the optimal hyperplane can be written as:*wx^T^*+*b* = 0, where *w* is the weight vector, and *b* is the bias. The nearest points on each side of optimal hyperplane are called support vector, for which |yi|⁢(w⁢xiT+b)=1. This distance between the hyperplane and the support vectors is called “margin,” which can be termed as $\frac{1}{{||w||}^{2}}$ and the objective of SVM is to find the appropriate *w* and *b* so that $\frac{1}{{||w||}^{2}}$ is maximal.

**FIGURE 4:**
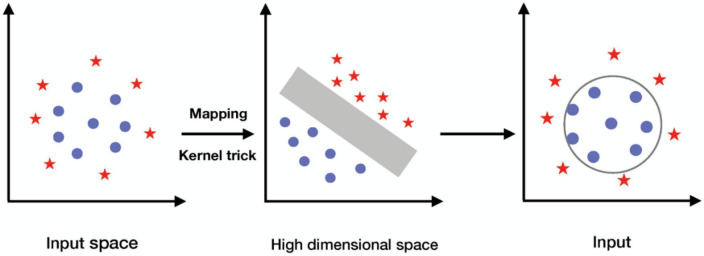
The procedure of using kernel to project the input data into higher, linearly separable dimension.

If the data is linear, hyperplane can be used to separate it. However, the data is frequently not linear. To facilitate this, kernels are utilized to add additional dimensions to the raw data, thereby transforming the problem into a linear one in the resulting higher-dimensional space. The SVM model is generated by mapping the input vectors from *d* dimensions to *f* dimensions space, denoted as ϕ:*R^d^*→*H^f^*, where *d* < *f*. Then, a kernel function *K*(*x*_*i*_,*x*_*j*_), which is the product of input vectors *x_i_* and *x_j_*, is used to construct an optimal separating hyperplane in the new feature space, where *K*(*x*_*i*_,*x*_*j*_) = ϕ(*x*_*i*_)⋅ϕ(*x*_*j*_) (27). As shown in Figure 4, the scalar product in input space is projected into a higher dimension with the kernel function, and the data become separable in the projected space.

Linear, polynomial, Gaussian, and Sigmoid are examples of frequent kernels. SVM has a number of hyper-parameters, and the optimal hyper-parameter can be found by trying possible combinations and observing which parameters work best. We found that the optimal hyperparameter is {*C* = 50, kernel = “rbf,” gamma = 0.00001}.

## 3. Results

### 3.1. Experimental results of orbital fat pad area segmentation

Here, the Orbital fat pad region segmentation using the U-net model was evaluated. The datasets, the experimental setting, and the results of our research are all described.

We manually mark 515 face images that have completed alignment and cropping as above. After marking, every image has two labeled areas–orbital fat pad area and background. Transferred the labeled image to mask image that the pixel value of the orbital fat pad areas in labeled parts is set to 255 and the pixels for the rest of the areas are set to 0. Randomly divide the data set into training and test sets in the ratio of 8:2.

To train the U-net model, we employed the PyTorch deep learning toolkit in Python. To train the model, the batch size was set to 1, the epoch to 50, and BCELoss was used for the loss for training the model. We used the Root Mean Square Prop (RMSProp) optimizer (learning rate = 0.00001, weight decay = 1 × 10^–8^, momentum = 0.9). The type of GPUs we used for training is GeForce RTX 2080 Ti. Of the 515 images obtained for orbital fat pad area segmentation, we randomly selected 80% for training and 20% for testing.

The accuracy of orbital fat pad area segmentation can be assessed by several criteria. The Dice coefficient and IOU evaluation criteria were used in this case Table 3. Shows the accuracy of orbital fat pad area segmentation.

**TABLE 3:** The evaluation criteria of the orbital fat pad region segmentation.

Criteria	Percentage (%)
Dice coefficient	88.3
IOU (eye)	79.7
IOU (background)	97.3
mIOU	88.5

### 3.2. Experimental results of NRS-2002 score classification

We take the samples with NRS-2002 scores less than 3 as the positive class and those with NRS-2002 scores greater than or equal to 3 as the negative class. In this study, we utilized 300 × 300 pixel sample images of the orbital fat pad region, which were generated by merging the mask image of the orbital fat pad region with the aligned, cropped facial image, to create our training and testing datasets. Specifically, the training dataset comprised 293 positive and 88 negative images, while the testing dataset consisted of 99 positive and 35 negative images.

For both training set and testing set, after extracting features using HOG, it is important to standardize them before using PCA to reduce dimensionality. Once the features have been standardized, PCA can be used to identify the principal components that explain the most variance in the data. These principal components can then be utilized as new features for training and testing. The processed features of training set were then fed into the SVM for training. After the end of training, the processed features of the test set were then fed into the trained SVM model to predict the NRS-2002 score, then examinate their classification accuracy, recall, precision, and F-measure rates, following evaluation criteria are used as given in Eqs. (1)-(4):


(1) $$a\,c\,c\,u\,r\,a\,c\,y=\frac{T\,P+T\,N}{T\,P+T\,N+F\,P+F\,N}$$

(2) $$p\,r\,e\,c\,i\,s\,i\,o\,n=\frac{T\,P}{T\,P+F\,P}$$

(3) $$r\,e\,c\,a\,l\,l=\frac{T\,P}{T\,P+F\,N}$$

(4) $$F_{1}=\frac{2\times p\,r\,e\,c\,i\,s\,i\,o\,n\times r\,e\,c\,a\,l\,l}{p\,r\,e\,c\,i\,s\,i\,o\,n+r\,e\,c\,a\,l\,l}$$

Table 4 shows the results of accuracy, recall, precision, F1-score of the proposed classifier for NRS-2002 detection.

**TABLE 4:** Results of accuracy, recall, precision, F1-score of the proposed classifier for NRS-2002 detection.

Criteria	Percentage (%)
Accuracy	73.1
Recall	93.9
Precision	97.3
F1-score	75.6

We also detect classification accuracy rates according to age. We conducted separate accuracy analyses of 20 elderly individuals and 114 non-elderly individuals in the test set. The results revealed that the accuracy rate for the elderly group was 85%, while the accuracy rate for the non-elderly group was 71.1%. We obtain *P* = 0.3056 from chi-square test, indicates there is no statistically significant difference in classification accuracy between elderly group and non-elderly group Table 5. Shows the prediction accuracy for categorizing Individuals as elderly or non-elderly.

**TABLE 5:** Prediction accuracy for categorizing individuals from age, gender, and location of hospitals.

	Correct prediction	Incorrect prediction	Total	Accuracy	P
**Age**					
Elderly	17	3	20	85%	0.306
Non-elderly	81	33	114	71.1%	
**Gender**					
Male	54	24	78	69.2%	0.315
Female	44	12	56	78.6%	
**Location**					
Remote	39	12	51	76.5%	0.629
Non-remote	59	24	83	71.1%	

For different genders Table 5, also shows the prediction accuracy for categorizing individuals as male or female. The classification accuracy rate for males is 69.2%, for female, the accuracy rate is about 78.6%. *P* > 0.05 indicates there is no statistically significant difference in classification accuracy between male and female.

Comparison based on the address location of the hospitals in the sample collection Table 5, indicates for hospitals in remote areas such as Tibet and Yunnan, the classification accuracy rate of collected patient samples is 76.5%; for hospitals in non-remote areas, the classification accuracy rate is 71.1%. *P* > 0.05 indicates there is no statistically significant difference in classification accuracy between samples from remote and non-remote location of the hospitals.

## 4. Discussion

In our study, we developed a machine learning model to predict nutritional risk using facial images. This model encompasses a series of algorithms, including image normalization, which adjusts pixel values to a standard scale; image segmentation, which partitions the image into meaningful regions; and the support vector machine (SVM) algorithm, a supervised learning method for classification and regression. Currently, the model’s accuracy is 73.1%, indicating moderate to high correlation with the NRS-2002 nutritional risk scores.

The achieved accuracy of 73.1% holds significant implications for the feasibility of this method. Although not perfect, this level of accuracy showcases the potential for further improvements. The development of such an algorithm has the potential to revolutionize nutritional risk assessment by providing a non-invasive, cost-effective, and easily accessible tool for healthcare professionals and individuals alike. This method could enable rapid screening of large populations, helping to identify those at risk of malnutrition more efficiently, and subsequently allowing for early intervention and better patient outcomes. Furthermore, the integration of AI-based nutritional risk assessment tools can reduce the need for extensive training and eliminate the subjectivity associated with traditional assessment methods.

In the context of policy-making, the adoption of AI-based nutritional risk assessment tools could facilitate more informed decisions regarding insurance reimbursement policies (28). By providing an objective measure of nutritional risk, insurance providers can better allocate resources and tailor reimbursement policies to meet the needs of patients with varying degrees of malnutrition. This could lead to more equitable distribution of resources and improved patient care.

With respect to clinical practice, the use of AI-driven facial image analysis for nutritional risk assessment can streamline the process, enabling healthcare professionals to make more informed decisions about patient care (29). This technology can complement existing assessment methods, providing a more comprehensive understanding of a patient’s nutritional status. Additionally, the real-time nature of the analysis can help clinicians monitor changes in a patient’s nutritional status over time, allowing them to adapt treatment plans accordingly.

As for industry development, the integration of AI-based nutritional risk assessment tools could drive innovation in the field of digital therapeutics (30). Companies could leverage this technology to develop new products and services, such as mobile applications and telehealth platforms, that utilize facial image analysis for nutritional risk assessment. These applications could extend the reach of nutritional screening services, particularly in remote or underserved areas, and empower individuals to take a more active role in managing their own nutritional health.

Our research contributes to the existing body of knowledge in several ways. First, we demonstrate the potential of using facial images for nutritional risk assessment, effectively bridging the gap between AI technology and clinical nutrition. Second, we provide valuable insights into the feasibility of using machine learning models for predicting nutritional risk scores, which can serve as a foundation for future researchers to optimize and refine.

Despite its contributions, our study has certain limitations. One limitation is that we only established the relationship between orbital fat pads and NRS-2002 scores. Future research can expand the model to incorporate other nutritional indicators and digital biomarkers, such as temporalis muscle thickness, masseter muscle thickness, and other facial features. This would enable a more comprehensive assessment of nutritional risk based on a variety of facial characteristics. Another limitation is the insufficient amount of data used for model development, which may have affected the model’s performance.

Future researchers can address these limitations by developing new models and validating their effectiveness. This may involve exploring different machine learning algorithms, incorporating additional digital biomarkers, and increasing the sample size to improve the model’s performance. Moreover, researchers could investigate the role of demographic factors, such as age, gender, and ethnicity, in the relationship between facial features and nutritional risk.

In summary, the development of an AI-based nutritional risk assessment tool using facial image analysis has the potential to bring about transformative change in the healthcare sector. By enhancing the efficiency, accessibility, and objectivity of nutritional risk assessment, this technology can contribute to improved patient care, more equitable resource distribution, and the development of innovative digital therapeutics. As the accuracy and performance of these tools continue to improve, their widespread adoption can lead to significant advancements in policy-making, clinical practice, and industry development.

Further advancements in AI-based nutritional risk assessment tools may have far-reaching implications for various stakeholders in the healthcare ecosystem. For example, healthcare providers can benefit from more efficient and accurate patient assessment processes, enabling them to allocate resources more effectively and deliver tailored interventions. Patients, on the other hand, can experience better healthcare outcomes due to early detection and intervention of malnutrition. Moreover, these tools can improve public health strategies, enabling policymakers to make data-driven decisions and implement targeted nutritional programs for at-risk populations.

## 5. Conclusion

In conclusion, the integration of AI and facial image analysis for nutritional risk assessment represents a promising avenue for future research and development. As we continue to refine these models and address their limitations, the potential of this approach to revolutionize nutritional screening and positively impact the healthcare sector will only grow. The advancements in AI-based nutritional risk assessment tools can facilitate significant improvements in policy-making, clinical practice, and industry development, ultimately leading to better healthcare outcomes for patients and communities worldwide.

## Data availability statement

The datasets presented in this article are not readily available because of ethical, legal, and privacy concerns. Requests to access the datasets should be directed to hechengyuan@rplushealth.com.

## Ethics statement

The studies involving human participants were reviewed and approved by the Ethics Committee of Yunnan Cancer Hospital and Ethics Committee of West China Hospital, Sichuan University. The patients/participants provided their written informed consent to participate in this study. Written informed consent was obtained from the individual(s) for the publication of any potentially identifiable images or data included in this article.

## Author contributions

JW conducted the experiment and wrote the first draft of the manuscript. ZL had substantial contributions to the design of the work and revised the manuscript critically for important intellectual content. CH wrote some sections of the manuscript and critically revised the manuscript. All authors contributed to the article and approved the submitted version.
